# State of the Art Techniques for Water Quality Monitoring Systems for Fish Ponds Using IoT and Underwater Sensors: A Review

**DOI:** 10.3390/s22062088

**Published:** 2022-03-08

**Authors:** M. Manoj, V. Dhilip Kumar, Muhammad Arif, Elena-Raluca Bulai, Petru Bulai, Oana Geman

**Affiliations:** 1 Vel Tech Rangarajan Dr. Sagunthala R & D Institute of Science and Technology, Chennai 600062, India; smrtmano@gmail.com; 2 Department of Computer Science and Information Technology, University of Lahore, Lahore 54590, Pakistan; arifmuhammad36@hotmail.com; 3 Faculty of Medicine and Biological Sciences, Stefan cel Mare University, 720229 Suceava, Romania; bulairaluca@usm.ro; 4 Faculty of Mechanical Engineering, Automotive, and Robotics, Stefan cel Mare University, 720229 Suceava, Romania; bulaipetru@usm.ro; 5 Faculty of Electrical Engineering and Computer Science, Stefan cel Mare University of Suceava, 720229 Suceava, Romania

**Keywords:** Arduino, Internet of Things, pH sensor, sensors, water quality monitoring system

## Abstract

One of the major issues facing the world is the resource of safe water, which is decreasing rapidly due to climatic changes, contamination, and pollution. The most affected living beings are underwater life forms as they eventually take these toxins in and are thus prone to death, making continuously checking water quality a quintessential task. But traditional systems for checking water quality are energy-consuming, involving the initial collection of water samples from different locations and then testing them in the lab. One emerging technology, the Internet of Things (IoT), shows great promise related to this field. This paper presents a detailed review of various water quality monitoring systems (WQSN), using IoT, that have been proposed by various researchers for the past decade (2011–2020). In this instance, new calculations are made for potential clients to analyze the concerned area of research. This review acknowledges key accomplishments concerning quality measures and success indicators regarding qualitative and quantitative measurement. This study also explores the key points and reasons behind lessons learned and proposes a roadmap for impending findings.

## 1. Introduction

**Figure 1:**
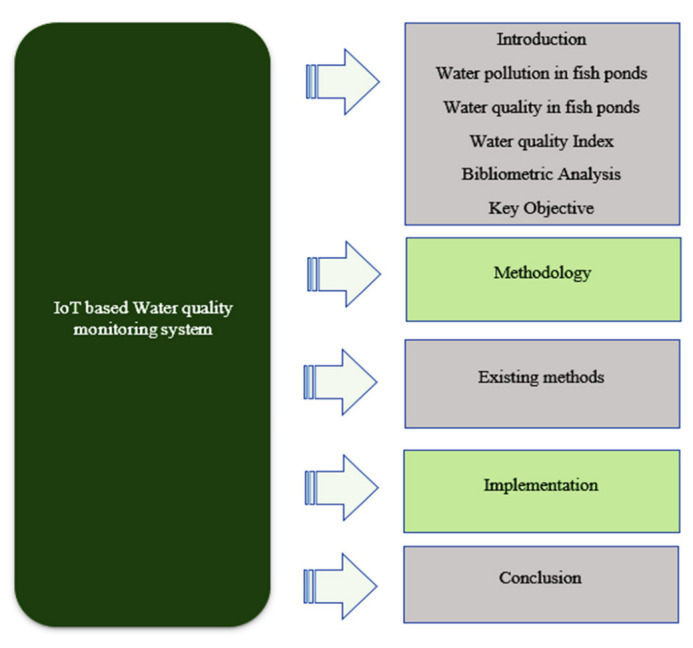
The overall structure of this paper.

This paper offers an effective IoT-based WQMS for fish ponds. Figure 1 depicts the structure of this paper.

**Figure 2:**
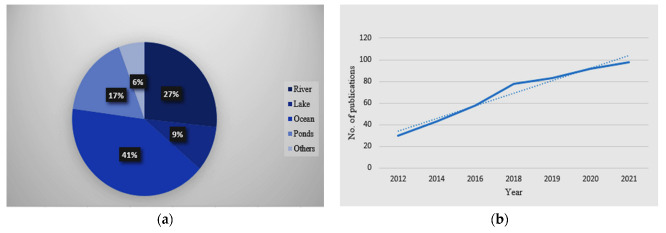
(**a**). Percentage of water pollutions in various water bodies, (**b**). No. of publication vs. year over water quality in water bodies.

The use of the Wireless Sensor Network (WSN), Online Water Quality Monitoring System, and Portable was proposed by Taufik Ibnu Salim et al. [1]. The quality and Leakage via the Detection of Water Level is an idea incepted by Arjun K et al. [2], using Raspberry PiTM along with the Internet of Things according to Sajith et al. [3]. The raspberry pi^TM^ and sensors relied on the proposed system. Water is one of the most essential elements for survival. The total volume of fresh water available for human usage and consumption is as little as 2%, though 70% of the Earth is covered by water (Yang et al. [4]). The human community faces a scarcity of water for consumption as the population rises, and the meager amount available is increasingly polluted by uncontrolled urbanization and industrialization. Of the many environmental challenges, the most pressing is the quality and availability of fresh water resources, though there are few areas containing potable water that pollution has left pure for monitoring. Several works analyzing water quality have been done. To verify and monitor the water quality in a large area, this paper presents a sensor node measurement device mainly focused on monitoring. Data are collected into the PMS database and displayed in real-time using a wireless sensor network. The consumers and authority’s database sets the values measuring the level, turbidity, pressure, and pH, stores the measures based on the threshold values, and sends notifications out (Lambrou et al. [5]). Figure 2a,b depicts the percentage of water pollution in the various water bodies and the number of papers published per year concerning water quality in water bodies. In Figure 2b, the dotted line is the linear fit, and the dark line shows the progression of papers published over the years by Effects of Water Pollution (NRDC [6]).

### 1.1. Water Pollution in the Fish Pond

The spread of disease caused by the presence of bacteria, algae, protozoa, and fungi in a fish pond can cause biological pollution and reduce fish product production. Winfield and Gerdeaux [7] say the fish infections were caused by primary pathogens such as Coliform bacteria and *E. coli*.

Fecal Coliform Bacteria: The bacteria causing different diseases in fish are grouped under Coliform bacteria (Gregor [8]). Their concentration is dangerously high in those fish ponds receiving animal waste or human waste from wildlife and septic systems, according to Garcia and Beıcares [9]. Coliform bacteria may also be introduced into small ponds by a large number of waterfowl, according to Burhanettin et al. [10]. A water test for fecal coliform bacteria can identify their presence and number of colonies per liter (Cho [11]). Only certified laboratories are recommended for this test. A concentration of less than ten bacteria per 100 mL of water is the optimum condition for fish farms (Annie et al. [12]).

***E. coli:*** *E. coli* is a more dangerous group of bacteria than coliform bacteria (Salamon et al. [13]). *E. coli* is fatal in small quantities, and the pond used for fish production must be *E. coli* free as their presence spreads serious disease and causes death (Apha [14]). Water tests are required for *E. coli* to identify their presence in respective ponds (Witte [15]).

Eutrophication: Excessive plant growth resulting from nutrient enrichment due to human activity is known as eutrophication. Some authors reported that eutrophication favored fish growth. Recent studies conclude that growth patterns with density dependence were more complex than once thought. The uncontrolled growth of algae or cyno-bacteria in fish ponds harms fish production and their habitat. Its impact is explained by Winfield J. and Gerdeaux D. [7], in which they give an example of fish death due to eutrophication in England and Scotland. Fish abundance and functional composition are directly related to the level of eutrophication. With the contribution of some fish species to the process, researchers succeeded in combating eutrophication or reoligotrophication through falling nutrient levels and re-gaining the original level (Asim et al. [16]).

Acidification: All scientists studying ecology agree that acidification due to various reasons negatively affects the water ecosystem, especially the freshwater ecosystem. Acid is the major contributor to the acidification of fish ponds. Massive damage to the S. *trutta* population in England and Scotland was documented. This research inspired a large-scale program to resolve this problem. Following this research, the recovery of water quality was reported, and S. *trutta* populations also recovered, as per Asim et al. [17].

PH: PH is the measure of water acidity. The PH value of pond water has significant importance in fish culturing (Berg and Fiksdal [18]).

Chemical Pollution: Chemical pollution is the same as acidification, though within a restricted distribution area. Industry plays a significant role in increasing environmental chemical pollution. Chemical pollution is dangerous and causes disease because the population has not developed immunity against chemicals (Hossain [19]. In the latter half of the 20th century, the major environmental problem received significant research attention showing that widespread pollution greatly affected fish production.

Pesticides and Herbicides: Used to control plant and algae growth, pesticides and herbicides introduced into the surrounding area contaminate the fish pond. (Waterfowl death and animal sickness are caused, and pesticide pollution kills fish) as per Schindler [20]. Heavy rain and wind following pesticide application to a nearby field may aid the pesticide in reaching the fish pond (Holopainen and Oikari [21]). The use of herbicides in the fish pond meant to control plants and algae must follow the herbicide’s label instructions (Michael et al. [22]). For irrigation purposes, pesticides used in water cause plant injury and damages the population badly, as the fish are not immune to the chemicals (Annett [23]).

Hardness and Metals: Water hardness is caused by the presence of magnesium and calcium in the water (Engel [24]). For fish, water hardness is not dangerous. Pond water in limestone areas is commonly hard (Wilde et al. [25]). In controlling pond plants and algae, the effect of herbicides used can be spoiled at a hardness above 50 m^2^/L (Clarke [26]), and the efficiency of copper-based pesticides can be reduced (Ahmad et al. [27]). An offensive taste develops in the inhabiting animals and aquatic environments contaminated with copper, manganese, and iron (Aboul-Ezz and Abdel-Razek [28]). In fish ponds located in coal mining areas, the concentration of iron and manganese is high (Waqar [29]). The pond’s aesthetic can be changed but did not cause disease in fish by these metals (Oguzie [30]). Above 0.3 mg/L, due to a high concentration of iron, orange precipitation may occur in the pond. Using copper-based herbicides for plant control in fish ponds with above 1 mg/L copper concentration can cause an offensive metallic taste (Norman [31]).

### 1.2. Safe Water Quality Parameters

Electric Conductivity: Produces electric current and aqueous solutions. It is generally used to measure the quality or degree of water. The safe limit is 300–800 µS/cm.

pH: Hydrogen-ion concentration is effective pH = −log[H+]. The safe limit is 6.5–8.5 pH.

Turb: With light transmission which hurdle solids suspended in water. The safe limit is 1–5 NTU.

ORP: Electrons via a chemical reaction to either accept or release the ability. The safe limit is 650–700 mV

Free-CI: In water to chlorinate microbes, it is responsible. The safe limit is 0.2–0.5 mg/L

RC: After chlorination which remains in the water, residual chlorine is the amount of chlorine. The safe limit is 2–3 mg/L

### 1.3. Water Quality in a Fish Pond

Water can quickly lose its ability to support life, reproduction, waste excretion, growth, and feed the fish in fish ponds. The needs of the fish, the water quality, and factors for managing the water quality should be understood by those wishing to be successful fish farmers. In filling their fish ponds with water, farmers should pay attention to chemical and physical aspects (Lucy [32]; Water Pollution Facts [6]; Groundwater [33]).

Temperature: Feeding, growth, and reproduction can affect fish welfare, and controlling the temperature is very important. Purdue University says that for each −7.8 °C rise in temperature, the metabolic rates in fish will double. Optimal fish growth varies depending on its optimal average temperature range; on whether the fish is cold-water, cool water, or warm water, and the temperature of the water depends on the availability of the fish selected for the pond. The optimal temperature range for the growth of cold-water trout and salmon is between 09–18 °C. Catfish and tilapia are warm-water fish that prefer temperatures between 24–27 °C, while yellow perch are cool-water species that prefer between 15 °C and 30 °C (EPA [34]; Ezugwu et al. [35]).

Suspended solids: Recirculating aquaculture systems cause water problems related to clay suspended particles, along with plankton, fish wastes, and uneaten feed. In these systems, up to 70 percent of the fish waste nitrogen load may contain particles representing a major source of irritation to fish gills. Fish, as a rule, produce one pound of waste per each pound of body weight (Ahmed et al. [36]; Cloete et al. [37]; Theofanis P. Lambrou et al. [5]).

Photosynthesis: Photosynthesis is the process by which food source carbon dioxide is converted. As a byproduct, and using sunlight, the oxygen is released into phytoplankton. In fish ponds, nitrogenous wastes such as ammonia, nitrates, and urea remove several forms of photosynthesis. The greatest concentrations of the photosynthetic process, driven by oxygen occurent sunlight, usually occur from 2–3 pm. Phytoplankton are primary respirators. At night photosynthesis ceases (EPA [34]; Brands et al. [38]; Loganathan et al. [39]; Geetha and Gouthami [40]; Abba et al. [41]).

Dissolved Oxygen: Directly or indirectly, dissolved oxygen (DO) is the most important chemical parameter in aquaculture. Low-dissolved oxygen levels are responsible for more fish deaths than all other problems combined. As it is with human respiration, fish require oxygen. The activity level, size, feeding rate, and temperature of the fish affect the amount of oxygen it requires. Lewis et al. [42] determined that, per day, striped bass consumed 0.012–0.020 pounds per pound at 25 °C. A temperature increase for each −7.8 °C, which doubles the metabolic rate of a striped bass, may be due to the higher oxygen requirement. Concerning increases in altitudes at higher temperatures, decreases, and salinities, Table 1 depicts the DO amount in water in which the water decreases the amount of oxygen that can be dissolved.

***CO_2_:***CO_2_ originates from limestone-bearing rock in water sources or photosynthesis. Fish can tolerate dissolved oxygen concentrations of 10 ppm. Good water supporting a carbon dioxide-free environment for fish inhabitants normally contains less than 5 ppm. From noon to daybreak at 5:15 a.m. in an intensive pond fish culture, carbon dioxide levels in water may fluctuate (Pule et al. [43]; Adu-Manu et al. [44]).

***Nitrogen:*** Dissolved gases, especially nitrogen, are usually measured in terms of “percent saturation”. The water normally holds the amount of gas saturation at a given temperature. A gas above 110% supersaturation level is usually considered problematic.

***Ammonia:*** As wastes enter the water, the amount of urea and fish excretion ammonia lessens. The ionized and un-ionized ammonia in aquaculture systems occur in two forms. The ionized form (NH4+) is not toxic ammonia. NH3 is very toxic in un-ionized form. Both forms of “total ammonia” are grouped. To harmless nitrates, toxic ammonia can be degraded through biological processes. Table 2 depicts a pH increase as temperature un-ionized ammonia levels rise.

### 1.4. Water Quality Index (WQI)

Drinking water to compute the WQI of USEPA and WHO recommend WQM parameters concerning different living conditions around the globe (Kashid et al. [45]; Chowdury et al. [46]; Kumar et al. [47]; Kawarkhe et al. [48]; Prasad et al. [49]). WQI parameters show that measuring water quality by traditional laboratory-based methods is commonly utilized.

Total coliform (TC): In soil, human and animal waste, etc., bacteria are usually present. Generally, humans and animal feces contain a class of TC belonging to the fecal coliforms. TC measuring methods commonly use minimal medium ONPG and multiple tube fermentation, numbering the most probable membrane filtration. Organisms/100 mL is its unit. The disease-causing pathogen is a signal to humans, but its presence in Coliform bacteria is usually harmless. Gastrointestinal upset and general flu-type symptoms (e.g., abdominal cramps, fever, and diarrhea) are commonly observed symptoms.

Fecal coliform (FC): Of total coliform, it is a subdivision. Escherichia coli (E-Coli) is the most common member. In humans and animals both warm and cold-blooded, bacteria exist in waste and intestines. Other pathogenic organisms may exist, but FC are not pathogenic by themselves. ONPG is a common method of measuring FC, recording the most probable number, membrane filtration, multiple tube fermentation, and minimal medium. Organisms per 100 mL number are its measuring unit.

Total dissolved solids (TDS): Magnesium, calcium, sodium, potassium cations, etc., present in the water represent soluble solids both organic and inorganic. A minimum threshold, if it increases, becomes saline beyond salinity because the water is highly correlated. Fertilizers, pesticides, sewage treatment, floodwater, etc., are the major sources. To measure it in mg/L, the gravimetric method is generally used.

Total suspended solids (TSS): In water, both organic and inorganic material suspended represents the number of remains. Light absorption is correlated. Let water absorbs less oxygen, and more light absorption may increase TSS. This may have adverse effects on aquatic life. In mg/L to measure it is generally utilized the gravimetric scheme.

Total solids (TS): Suspended solids represent the total amount of solids when water is dissolved. Sulfur, calcium, phosphorous, nitrate, iron, etc., are generally dissolved solids. Plankton, algae, silt, clay particles, etc., may be included. The aquatic plant process affects photosynthesis in turn; the passage of sunlight through water can affect water clarity. Retaining more heat may adversely affect aquatic life, and water will heat up due to this.

Total hardness (TH): For domestic or industrial applications, TH determines the suitability of water. In water, the presence of magnesium and calcium is the concentration. With an EDTA solution, it is generally measured using a titration method. In mg/L or parts per million (PPM), calcium carbonate (CaCO_3_) hardness is given in terms of equivalent quantity. Magnesium and calcium are basic hard water minerals that can fulfill dietary needs, that may be beneficial for humans but are not harmful. The heated formation of calcium carbonate is the major drawback of hard water, leaving decay deposits on heating elements and pipes.

Dissolved oxygen (DO): In water, oxygen solubility is represented by DO gained from the atmosphere during photosynthesis or absorbed generally from the water. For aquatic life, it plays an essential role. It corrodes water pipes, but it may make water taste better for drinking. It is highly important for aquatic life. For example, aquatic life undergoes stress when its level falls below 5 mg/L. An electric meter or Winkler titration is generally utilized for measurement purposes.

Electrical conductivity (EC): EC represents water’s ability to conduct electric current. Water’s ionic content helps with measuring alkalinity, hardness, and some dissolved solids, though it is not involved directly. Measurement methods utilized are specifically electrical.

Chloride (Cl): Water is measured using the mg/L titration method (milligram per liter is naturally available). If 250 mg/L is a minimum threshold, exceeding it may make water taste saltier though the excess may not damage humans. For agricultural activities, excessive Cl may be harmful. Due to corrosiveness, the electrical conductivity of water increases, reacting due to soluble salts forming with metal ions in metallic pipes. This also raises the level of metals in water.

Temperature (T): T affects the chemistry of water. It increases at higher temperatures because of chemical reactions. At higher temperatures, groundwater especially can dissolve more minerals from rocks surrounding the water. Electrical conductivity will increase due to this act. Rates of gas transfer affect dissolved oxygen and have a great effect on aquatic life. It is often measured in Celsius.

Potential of hydrogen (pH): Normal water has a pH of 7. Alkalinity means a range from 8 to 14, while acidity indicates a value from 0 to 6. For humans, water with pH values from 6.5 to 8.5 is generally safe to drink [17]. Electrodes and electrometry are measured using pH. If corrosive and soft, then the water is acidic.

Oxidation-reduction potential (ORP): Also known as REDOX, it is a millivolt (mV) measurement to determine either reduction or oxidization substance capability. To measure ORP, an ORP meter is used. A positive reading means the substance is an oxidizer (i.e., acceptor of electrons). A negative reading means a reducer (i.e., donor of electrons). A high ORP generally having chlorine, it is added to water to kill unwanted bacteria and pathogens. Bacteria’s DNA, and proteins from cell membrane oxidation, will attract electrons. In addition, it can disinfect water oxygen, which also has high ORP.

Total chlorine (T-Cl): This represents the levels of free and combined chlorine. To measure free chlorine and maintain residual levels, it is necessary to add appropriate solutions. To kill harmful microorganisms (e.g., viruses and bacteria), numerous municipalities intentionally add chlorine to water which, if ingested, could make us sick.

Free chlorine (F-Cl): Residual, chlorine residual, or residual chlorine is well known. Water level potability is indicated. As a dissolved gas, (Cl_2_), hypochlorite ion (OCl−), and/or hypochlorous acid (HOCl) is the amount of residual chlorine (RC) present in the water. A test kit can measure the total amount of Cl_2_, OCL, and HOCL. The measurement unit is mg/L. It is generally used to disinfect contaminated water. In digital water colorimeters or color-wheel test kits, F-Cl is tested via pool test kits. Free from recontamination during storage and most disease-causing pathogens means water is protected from its presence.

### 1.5. Bibliometric Analysis

**Figure 3:**
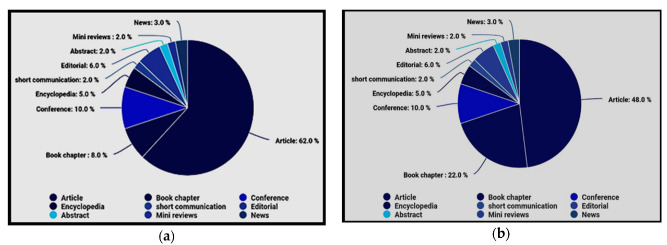
(**a**) Percentage-wise cluster over keyword “Water Quality Monitoring System using IoT”. (**b**) Percentage-wise clusters using keyword “Smart Water Quality Monitory System”.

This paper presents a detailed review of various papers from the past decade taken from databases such as IEEE Xplore, Science Direct, MDPI, ASCE library, Copernicus, AAS, Springer, Science press, Oxford Academic Press, and Scopus, state-of-the-art models. In these databases, the keywords used for extracting these data are “Water Quality Monitoring System using IoT” or “Smart Water Quality Monitory System”. This particular search may impact directly or indirectly. A total of 4209 documents are carried from these 10 databases, clustered into certain categories over the past decade. While analyzing each database, the basic clusters obtained are Article (65%), Book Chapters (8%), Conference Papers (12%), Encyclopedia (3%), Short communication (2%), Editorial (2%), Abstract (2%), Mini review (2%), News (4%). From this, it is clear that for every section in each database that we collected, there is a certain weightage, and IEEE Xplore has the greatest number of publications. Figure 3a represents a percentage-wise cluster in 10 databases using the keyword “Water Quality Monitoring System using IoT”. Figure 3b shows percentage-wise cluster in 10 databases using Keyword—“Smart Water Quality Monitory System”.

### 1.6. Key Highlights

This paper depicts the effective ways to implement the Water Quality Monitoring System. The following are the key objectives:

- Reviewing the latest papers proposed by various researchers concerning this area for the past decade (2011–2020).
- Depicting the significance of IoT usage in Water Quality Monitoring Systems (WQMS).
- Quantitative examination through various measurements showing the viability and decency of ongoing plans.
- Mostly helpful for fish pond analysis as these systems will check quality and safeguard living beings inside the water.

***Organization of this paper:*** As we already came across the overview of water pollution and its several quality indexes in the introduction in Section 1, the rest is as follows; Section 2 depicts the methodology of WQMS with the help of IoT, Section 3 depicts the papers associated with this WQMS system proposed by various researchers, Section 4 depicts the implementation and analysis.

## 2. Methodology

**Figure 4:**
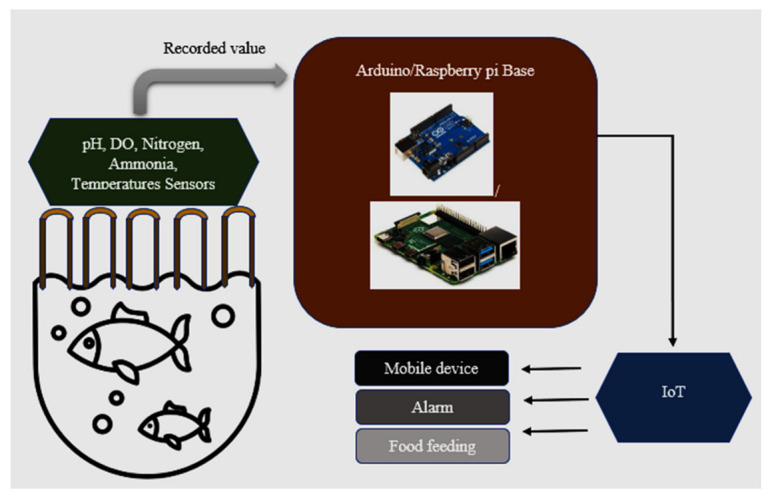
Proposed WQMS using IoT system.

The aim is to create a smart freshwater pond for aquaculture with automatic maintenance and food feeding systems (Figure 4). Our system also includes an automatic alert. For the fish to grow healthy, water quality needs to be maintained by maintaining its parameters. So, to maintain the water quality parameters, we are installing underwater sensors to continuously record the values of the parameters in the regular interval.

Parameters such as pH, dissolved oxygen, nitrogen, ammonia, and temperature determine water quality. We need sensors/IoT devices to monitor said parameters. The values recorded by the IoT devices are sent to Arduino/Raspberry-pi for processing. There, the comparisons are carried out, and the difference in value determines actions taken to maintain the desired values. The impartation to the farmer is sent through the cellular unit attached with Arduino. The sensors connected to the pond collect the data and send it to Arduino for processing, where the comparison occurs. This happens at regular intervals. Differences in the desired values need to be addressed. If the dissolved oxygen is not at the desired value, the aeration unit will work automatically as instructed by Arduino. If the water level is low, the motor will switch ON automatically. Also, we include an automatic fish feeder that automatically feeds the fish at regular intervals, avoiding over/underfeeding. This first stage implementation means a fully automated, human-less fish farming system. The water recycling system is included.

## 3. Existing Methods

For the past decade (2011–2021), research specialists have presented various research papers concerning this smart aquaculture. Table 3 depicts the overall summary of existing systems.

Ma et al. [50] proposed a system for scientific management based on the GIS of water quality information. It provides reference by taking information from the water quality management system and designs technological implementation. The platform gives the example of MapInfo in Fuzhou city in the Jiangxi province. A water quality monitoring system using wireless sensor network technology was proposed by Qiuchan et al. [51], addressing water quality requirements efficiently and intelligently. It reduces the impact of water pollution and prevents environmental water pollution by accurately monitoring data that affect water quality in real-time, acquiring multiple parameters, and monitoring online functions.

A combination of static-dynamic monitoring nodes achieves real-time online monitoring of the whole water environment, including water quality sampling at any designated location. This can be achieved via quality monitoring and sampling undertaken by an autonomous cruise ship, as presented by Shuo et al. [52]. They proposed an unmanned ship using the actual monitoring unit system, the ground station control unit system, and the control unit system to perform online, real-time water quality monitoring and sampling.

A remote system for efficiently and practically monitoring environmental water quality was proposed by Wang et al. [53]. It involves changes in the neural network grey-BP, and is based on the water bloom prediction method. Additionally, it monitors information automatically in real-time through wireless communication technology and provides water bloom early warnings based on GPRS. The system consists of the soil monitoring node, routing node, water quality monitoring, and gateway server.

A WSN source-based system for monitoring rural drinking water was introduced by Lin et al. [54]. The GPRS module unifies the uploaded data collection in the gateway node, or routes the wireless module to send it directly to the gateway node. To realize online monitoring for pollution control and to provide non-point source soil pollution information, the system periodically detects important indicators related to pollution guidance in rural water sources and soil. The pH error range is 0.64~1.68%, Cu concentration range of error is 1.98~2.22%, Cu concentration range of error is 1.58~2.01% for 1.09~1.86%. The acquisition error system facilitates data acquisition and remote transmission. The network test shows the stability range of the designed dissolved oxygen system.

Discharge and water quality standards for industry bring about syntaxis protection and utilization of water resources. To provide a reliable basis for water quality prediction, Zhang et al. [55] proposed the quality information be visually displayed. Jha et al. [56] designed a system to monitor water quality and usage in real-time using a Smart Water Monitoring System (SWMS). A Smart Water Quality meter and Smart Water Quantity meter are employed. The consumer and the authority monitoring a household are notified regarding the amount of water consumed to ensure water conservation. According to the quantity consumed, the billing system generates a three-slab. The Smart Water Quality meter checks the consumer’s water for pH, conductivity, temperature, turbidity, and dissolved oxygen, measuring potability and purity. This ensures that any health hazards or potential threats due to the accidental seepage of sewage or farm release are prevented. The cloud data provides for online monitoring in real-time. The system generates an SMS alert signal sent to both consumer and authority concerning usage limit, water quality, or to notify of violations immediately.

Hamid et al. [57] proposed controlling water quality in real-time to develop an affordable system. Conductivity, pH, turbidity, and the temperature of various chemical and physical water properties are measured via several sensors integrated into the system. IoT technology is used. The sensor that processes captured data, which can also be the microprocessor, manages the core controller. Cloud computing via the internet can accomplish visualization of data. Pang et al. [58] proposed, designed, and actuated a multiple water quality monitoring system based on SVM. To guarantee the effectiveness and timeliness of this system using the Gauss Radial Basis Function, and through offline studies of samples, it established a water quality corresponding evaluation model. In the paper, instance interface is also determined by classifying categories aside from corresponding water quality. According to the results of the Central Line Project of the South-to-North Water Diversion Project, the system is stable and effective, and this safe system has been successfully applied.

A mobile water quality monitoring system combining bionic underwater robot fish and wireless sensing technology was presented by Kai et al. [59]. The bionic system can minimize disturbance to the water environment. At multiple points and different depths, the robot fish (called RoboSea-2.0) performs water quality detection, its movements controlled by the host computer software. Tests showed the flexibility of water quality, stable data transmission, and conducted real-time underwater platform moves. In the future, it could have a profound application for facilitating water quality and statistical data analysis.

Daigavane et al. [60] presented the design and development of a low-cost system for real-time IoT monitoring of water quality. Physical and chemical parameters of the water are measured by a system consisting of several sensors. Water parameters such as PH, temperature, turbidity, and flow can be measured. The core controller sensors can process the measured values. An Arduino model was used as a core controller. Using a WI-FI system, the sensor data can be viewed on the internet. Pokhrel et al. [61] depict monitoring water in real-time using IoT low-cost monitoring system was proposed. Data on a platform of microcontroller system and GPRS are used in different sensors such as pH, turbidity, temperature, and communication. In this system, quality parameters are measured.

Various parameters exist in the surrounding atmosphere, such as tank water level, pH value, humidity, turbidity in the water, and temperature. A measuring system consisting of several sensors was proposed by Pasika and Gandhla [62]. A personal computer (PC) interfaced with a microcontroller unit (MCU) performs further processing using these sensors. The water quality is monitored using an IoT-based Think Speak application, and the obtained data is sent to the cloud. A water tank project for finding pH value using the Arduino board and the GSM module for messaging was implemented by Moparti et al. [63]. For monitoring water parameters, they used an LED display. Finally, the cloud is used to globally monitor the pH value of water, message the user, and send data.

Madhavireddy and Koteswarrao [64] proposed in the IoT with an increased device network method in the wireless by transmission, data acquisition, and method observation is examined a real-time water quality. WI-FI interface is used by the core controller ARM to measure values from the sensor’s microcontroller, and the values are remotely processed. Observation using IoT setting sensors with quality observation interface projected the water quality. Using multiple device nodes, the WQM selects parameters for water such as temperature, pH level, water level, and CO_2_. This methodology sends the information to the webserver. The data may be retrieved or accessed from the server at any place in the world, and the data are updated at intervals. A buzzer will be ON into abnormal conditions if the sensors do not work or get.

## 4. Implementation

In this paper, we implemented water quality management systems using IoT and Under Water Sensors. The requirements for implementation are mentioned in Table 4. As discussed in Section 2, the main parameters defining water quality are pH, DO, nitrogen, ammonia, and temperature. We used the Arduino/Raspberry pi board for processing and transferring data via sensors that measured these parameters.

The sensor records the data at regular intervals. The data is collected and sent to the Arduino/Raspberry pi board. Here the data will be compared to normal values, and if any change is found, the board will take the necessary action to make the parameter values normal.

Parameters such as temperature, pH, and dissolved oxygen can be measured directly using associated sensors. This can be normalized by performing water level maintenance via turning the motor on and switching on the aeration unit. A pH of 7 or under can be called an acidic environment, and above 7 an alkaline. The pH value is down due to acidic rain. Due to this, the fish cannot sustain itself and may die. The ammonia must be tested using the AmmoLyt, which senses the presence of ammonia in water. Nitrogen can be measured using a Nitrate Smart Sensor. 

### 4.1. Traditional WQMS vs. IoT Based WQMS

**Figure 5:**
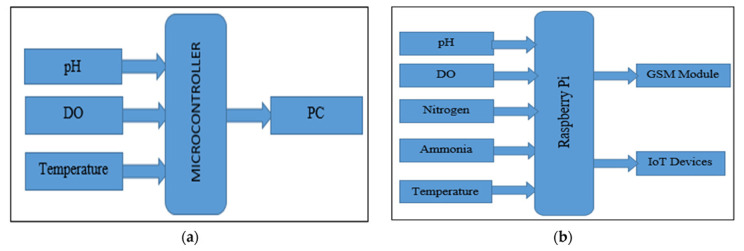
(**a**). Traditional WQMS (**b**). IoT based WQMS.

The Traditional WQMS system is shown in Figure 5a. In the traditional WQMS, all the sensors are connected to the microcontroller, and the data can be read through a personal computer attached to it. Whenever a mismatch is found in the data, further operations are done manually.

The IoT-based WQMS system is shown in Figure 5b. In the IoT-based WQMS, the sensors connected to the Raspberry Pi are connected to the IoT and GSM module for passing the information along to the farmers. The IoT maintains the correct values if any mismatch in the data is found.

This paper depicts an effective IoT-based water quality monitoring system for fish ponds. We highlighted the overview of water pollution and its overall increasing rate on water bodies throughout the year. The pollution that could enter fish ponds was depicted along with the parameters used for measuring and monitoring. Then we presented the bibliometric analysis of this topic using 10 popular databases, primarily the IEEE Xplore. We also briefly presented the implementation of WQMS, and depicted related proposal papers over the last decade (2011–2020). Finally, this will be useful for various research specialists to get new and innovative ideas to develop or integrate new technology to bring even more effective monitoring systems.

### 4.2. What Is New in This Article?

Though ample numbers of bibliometric analysis papers regarding smart water quality monitoring systems exist, this attempt covers papers regarding smart water quality monitoring systems over the past decade (2011–2020) with all possible algorithms discussed. This will aid in understanding the progress of quality monitoring systems over the past decade.

### 4.3. Futuristic Way to Multidisciplinary Research

As this paper mentions the story of smart water quality monitoring systems over the past decade, it will potentially help the fish farming and research communities enable better integration and the development of a model capable of even more accurate theoretical and practical results.

### 4.4. Bibliometric Inferences

This paper gives a brief account of various publications presented by active researchers. It also presents a brief review of various researchers who introduce great innovations for water quality monitoring systems, essential tools required to get a clear view of this field.

### 4.5. Limitations & Future Enhancements

As IoT has many security concerns compared to other new and evolving technologies like Machine Learning, Deep Learning, and many more, it can be used to upgrade security as well as overall system immunity in more effective ways.

## Figures and Tables

**Table 1:** DO amount in water.

Variable	Temperature (°C)				
	19	21	23	28	31
Salinity (ppm)					
0	9.4	9.1	8.7	7.9	7.5
5000	8.9	8.6	8.3	7.5	7.2
10,000	8.5	8.2	7.9	7.1	6.8
altitude (m)					
0 (Sea Level)	9.4	9.1	8.7	7.9	7.5
305	9.0	8.7	8.4	7.6	7.3
610	8.7	8.4	8.1	7.3	7.0

**Table 2:** un-ionized ammonia levels.

pH	15 °C	20 °C	25 °C	90 °C
7.0	0.25	0.4	0.6	1.0
7.4	0.6	1.0	1.5	2.4
7.8	1.6	2.5	4.0	5.7
8.2	4.1	5.9	10.0	13.2
8.6	8.4	13.7	20.7	27.7
9.0	19.6	28.5	39.1	49.0
9.2	38.3	50.0	61.7	70.8
9.6	60.2	71.2	79.4	85.9
10.0	72.4	79.9	85.6	90.6

**Table 3:** Overall summary of existing systems.

Authors	Technology	Baseboard	Sensors				
			pH	Ammonia	Temp	Nitrogen	DO
Ma et al. (2011)	IoT		✓		✓		
Qiuchan et al. (2020)	IoT	Arduino	✓		✓	✓	
Shuo et al. (2017)	IoT	Raspberry pi	✓		✓		
Wang et al. (2010)	DL	Arduino	✓		✓		
Lin et al. (2017)	IoT-WSN	Arduino	✓		✓		✓
Zhang et al. (2012)	IoT	Node MCU	✓		✓		
Jha et al. (2018)	IoT	Node MCU	✓		✓		✓
Hamid et al. (2020)	IoT-Cloud	Arduino	✓		✓		
Pang et al. (2013)	ML-IoT	Arduino	✓	✓	✓		
Kai et al. (2020)	AI		✓		✓		✓
Daigavane et al. (2017)	IoT	Arduino	✓		✓		
Pokhrel et al.(2018)	IoT	Arduino	✓		✓		
Pasika and Gandhla (2020)	IoT	MCU	✓		✓		
Moparti et al. (2018)	IoT	Arduino	✓				
Madhavireddy and Koteswarrao (2018)	IoT	MCU	✓		✓		

**Table 4:** The specification used for implementation.

No.	Hardware/Software Specification	Image	Description
1.	Arduino/Raspberry pi as baseboard	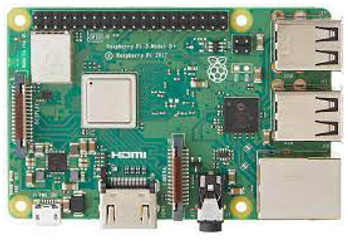	The Raspberry Pi is a low-cost, small-sized computer that connects to a monitor or TV and uses a standard keyboard and mouse.
		(or) 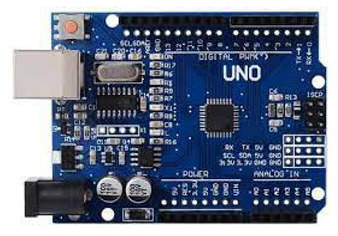	The Arduino is an open-source microcontroller developed by Arduino.cc. The board consists of digital and analog input/output (I/O) pins that may be connected/interfaced to various expansion shields. It is programmable with the Arduino Integrated Development Environment through a type B USB cable. It can be powered through a USB port or by an external 9-volt power source
2.	pH sensor	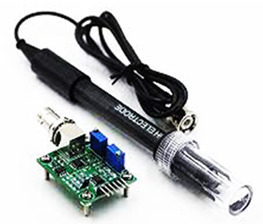	A pH sensor used for water measurements. Measures the amount of alkalinity and acidity in water.
3.	Temperature sensor (LM35)	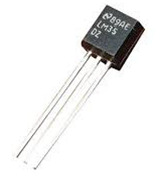	Reads the water temperature
4.	DO sensor	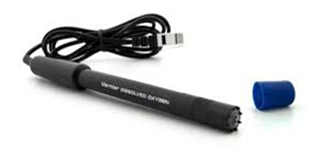	Used to measure the gaseous oxygen dissolved in water
5.	Nitrogen sensor	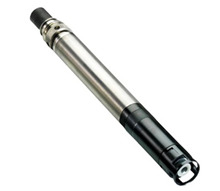	Sensor used to measure NO3 in freshwater applications
6.	Ammonia sensor	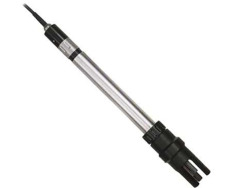	The AmmoLyt—used to detect the Ammonia Concentrations in water
7.	OS	Windows-10/Linux	Operating System to be installed in the computer that can be used for programming/configuring IoT devices.
8.	Mobile devices	Android/iOS	To support Mobile Applications and monitoring thereafter

## Data Availability

Not applicable.

## Abbreviations

AI	Artificial Intelligence
ML	Machine Learning
DL	Deep Learning
SVM	Support Vector Machine
WQMS	Water Quality Monitoring System
WSN	Wireless Sensor Networks
IoT	Internet of Things
DO	Dissolved Oxygen
CO2	Carbon Dioxide
TC	Total Coliform
WQI	Water Quality Index
FC	Fecal Coliform
TDS	Total Dissolved Solids
TSS	Total Suspended Solids
TS	Total Solids
TH	Total Hardness
EC	Electrical conductivity
CL	Chloride
T	Temperature
pH	Potential of hydrogen
ORP	Oxidation-Reduction Potential
T-CL	Total Chlorine
F-CL	Free chlorine
GPRS	General Packet Radio Service
GIS	Geographic Information System
MCU	Microcontroller Unit
PC	Personal Computer
WQM	Water Quality Monitoring
WI-FI	Wireless Fidelity
